# Vascular function in women: when studying perimenopause is *peri* important

**DOI:** 10.1113/EP091066

**Published:** 2023-02-20

**Authors:** Lyndsey E. DuBose, Wesley K. Lefferts

**Affiliations:** ^1^ Department of Medicine, Division of Geriatric Medicine University of Colorado Anschutz Medical Campus Aurora CO USA; ^2^ Department of Kinesiology Iowa State University Ames IA USA

**Keywords:** brain, menopause, oestradiol, vascular function

1

The menopausal transition is a time of accelerated cardiovascular ageing and increased susceptibility to chronic diseases including cardiovascular disease (CVD). Growing data support a cardiovascular contribution to brain ageing, but characterization of this in women remains unclear. In an article in this issue of *Experimental Physiology*, Ruediger et al. characterized group differences in peripheral and cerebrovascular function and their respective associations with sex hormones and nitric oxide bioavailability in healthy middle‐aged premenopausal (*n* = 10), early postmenopausal (*n* = 15) and late postmenopausal (*n* = 14) women (Ruediger et al., 2023). The authors should be applauded for conducting this comprehensive study and have taken an important step toward closing a critical gap in knowledge. Accordingly, we wish to highlight the importance of considering (i) the menopausal transition as a period of accelerated risk and clinical versus statistical significance, (ii) subject characteristics that may alter vascular ageing trajectories, and (iii) the paucity of data on cerebrovascular haemodynamics during menopause and importance of considering differing vascular methodology.

First, the menopausal transition is an inherently physiologically dynamic period. Cross‐sectional studies that exclude a portion of individuals (e.g., perimenopausal women) cannot account for the dynamic physiological changes during the menopausal transition. Cross‐sectional studies that exclude perimenopausal women also limit the distribution of hormones, reducing the ability to detect correlations between hormones and vascular outcomes. Gonadotropins begin to rise early in the menopausal transition prior to declines in oestradiol; however, these changes are accelerated around the final menstrual period. Increases in aortic stiffness are accelerated within 1 year prior to the final menstrual period, and progressive reductions in endothelial function begin in early perimenopause but accelerate in late perimenopause (Moreau et al., 2020; Samargandy et al., 2020). Experimental data suggest that oestrogen deficiency contributes to reductions in endothelial function with menopause and demonstrate a stronger association between follicle stimulating hormone and peripheral vascular function in women, suggesting additional mechanisms contribute (Moreau et al., 2020). These data are important to consider within the context of findings of Ruediger et al., which demonstrated no statistical differences in peripheral function between groups. Notably, the observed group differences in blood pressure (+5 mmHg), aortic stiffness (+0.4–2.2 m/s) and flow‐mediated dilatation (−1%) have been demonstrated to be clinically relevant, as noted by the authors, underscoring the need to consider clinical significance versus statistical significance, our second point of emphasis. Ruediger et al. also observed no differences in cerebrovascular haemodynamics and there are no established criteria for age‐related differences in cerebrovascular haemodynamics that have been linked to clinical risk to aid the interpretation of these data. Ultimately, Ruediger et al.’s data help address the overall paucity of data but should be interpreted cautiously because the modest sample size may limit statistical power to detect group differences of potential clinical significance.

Third, the limited literature examining cerebrovascular haemodynamics with menopause is inconsistent. Brislane et al., demonstrated that middle cerebral artery (MCA) velocity was lower in postmenopausal women compared with premenopausal women, but there was no effect of menopausal status on autoregulation or cerebrovascular reactivity found by Ruediger et al. (2023). Ruediger et al. also found no differences in shear‐mediated dilatation of the internal carotid arteries (ICAs), which conflicts with the study by Iwamoto et al. that demonstrated progressive reductions in ICA shear‐mediated dilatation across the menopausal transition (Iwamoto et al., 2021). Notably, differences in ICA shear‐mediated dilatation between menopausal groups were only present during the late follicular (high oestradiol) phase but not early follicular phase (low oestradiol) in premenopausal women (Ruediger et al., 2023). Ruediger et al. tested premenopausal women during the luteal phase when both progesterone and oestradiol are elevated but oestradiol is lower compared with the late follicular phase immediately prior to ovulation. Additionally, the effects of the menopausal transition on vascular function may differ by method of assessing cerebrovascular function (e.g., transcranial Doppler of MCA vs. CO_2_‐induced ICA dilatation vs. arterial spin labelling MRI with intravenous acetazolamide administration vs. functional or phase contrast MRI) and the level of the vascular tree (e.g., MCA vs. ICA vs. cerebral microvasculature). Indeed, regional cerebral microvascular blood flow, via arterial spin labelling MRI, is elevated in perimenopause potentially related to transient compensatory adaptations that shift substrate utilization, which may not be detectable when assessing larger cerebral arteries (e.g., MCA). These data highlight important considerations (i.e., menstrual cycle phase, methodology) that researchers should bear in mind when designing studies to investigate menopause and sex hormones on vascular function.

Finally, studies should comprehensively assess and report participant characteristics (i.e., physical activity, social determinants of health, menopause stage and symptoms) that may interact to alter vascular ageing trajectories across the menopausal transition and increase scientific reproducibility. In this study by Ruediger et al., 92% of participants met the Australian physical activity recommendations with the greatest levels among late postmenopausal women, potentially limiting the generalizability of the study. Physical activity is a key preventive behaviour to reduce CVD risk and recent data indicate sex‐specific benefits of fitness on large central artery (i.e., aorta and common carotids) and cerebrovascular haemodynamics in women, which may attenuate cross‐sectional differences in Ruediger et al.’s study (Lefferts et al., 2022). Women should be categorized according to the Stages of Reproductive Aging Workshop Survey, as performed by Ruediger et al. Future work should also measure and consider menopausal symptoms using established surveys like the ‘Menopause Symptom List’ developed by Janette Perz (Perz, 1997). Additionally, studies investigating cerebrovascular haemodynamics should measure apolipoprotein‐E4 (APOE‐4) status, a genetic risk factor for Alzheimer's disease, which further accelerates age‐related changes in biomarkers of Alzheimer's disease in perimenopausal and postmenopausal women (Mishra et al., 2021). Ultimately, we applaud the work by Ruediger et al. to address the paucity of data that exists regarding menopause‐related changes in the vascular contributions to brain ageing in women. Moving forward, researchers would be wise to pause and not overlook the ‘peri’ important role of the menopausal transition on vascular and brain health trajectories.

## AUTHOR CONTRIBUTIONS

Both authors approved the final version of the manuscript and agree to be accountable for all aspects of the work in ensuring that questions relating to the accuracy or integrity of any part of the work are appropriately investigated and resolved. All persons designated as authors qualify for authorship, and all those who qualify for authorship are listed.

## CONFLICT OF INTEREST

No competing interests declared.

## FUNDING INFORMATION

This work was funded by NIH F32AG071273 and Ludeman Family Center for Women's Health Research to L.E.D.
